# Community-based Disparities in the Treatment and Outcomes of Early-stage Non-small-cell Carcinoma

**DOI:** 10.7759/cureus.5889

**Published:** 2019-10-11

**Authors:** Shraddha Dalwadi, Bin S Teh, Eric Bernicker, E. Brian Butler, Andrew M Farach

**Affiliations:** 1 Radiation Oncology, Baylor College of Medicine, Houston, USA; 2 Radiation Oncology, Houston Methodist Hospital, Houston, USA; 3 Internal Medicine, Houston Methodist Hospital, Houston, USA

**Keywords:** access to care

## Abstract

Introduction

Lung cancer is the most common malignancy in men and women combined. It is also the leading cause of cancer-related deaths in the US. The objective of this study is to report the treatment and survival outcomes for early-stage non-small-cell lung carcinoma (NSCLC) when stratified by urban versus rural geography.

Methods

A dataset of 62,213 patients, all aged 60 years or above, with stage-1 NSCLC, who underwent treatment from 2004 to 2012 was retrieved from the Surveillance, Epidemiology, and End Results (SEER) database of the National Cancer Institute (NCI). Patients were divided into metropolitan, urban, or rural (in descending order of population density) based on their location of cancer treatment using the US Rural-Urban Continuum Code Definitions for 2003. Patient characteristics were compared using the chi-square test, and survival statistics were calculated using the Kaplan-Meier estimator.

Results

Rural or urban stage-1 NSCLC patients are more likely to be white, young, male, poor, and uninsured or Medicaid-dependent. They generally have squamous histology and receive radiation therapy when compared to metropolitan counterparts [probability value (p): <0.0001]. Median overall survival was shorter for rural and urban patients than metropolitan patients (41, 41, and 52 months respectively; p: <0.0001).

Conclusion

Tertiary care centers in metropolitan areas continue to demonstrate superior outcomes in the treatment of stage-1 NSCLC. This is presumably due to the existing disparities in patient access to care. Rural and urban stage-1 NSCLC patients (who tend to be younger, poorer, and more likely to be treated with radiation than surgery) are likely to be disproportionately impacted by changes in health policy.

## Introduction

Lung cancer is the leading cause of cancer-related deaths in the US and worldwide [1]. While most malignancies have seen an improvement in survival outcomes over the past decade, lung-cancer mortality rates remain poor, with a 5-year survival rate of only 18.6% [2]. This is partly due to most patients being diagnosed at an advanced, often incurable, stage. Recently, low-dose screening CT has been shown to improve survival in a high-risk cohort by shifting diagnosis to an earlier stage of the disease when multiple definitive treatment options exist [3]. However, the implementation of screening guidelines will inevitably lead to a consequential rise in early-stage diagnosis and increased public-health burden.

Geographic disparities and associated socioeconomic differences play an important and evolving role in cancer epidemiology [4-6]. In the 1950s to 1960s, more affluent and urban populations harbored worse cancer mortality rates. However, recent data up to 2007 show this relationship has not only reversed but that these differences have become more pronounced. Many factors contribute to geographic-area disparities, including differential comorbidity status and carcinogen exposure. Identification of high-risk groups and targeted intervention is key to developing sustainable improvements to cancer survival. This holds particularly true for lung cancer, where a shift to early-stage diagnosis should perhaps be accompanied by improved primary prevention and access to treatment.

Barriers to cancer care represent preventable morbidity and mortality in early-stage non-small-cell lung carcinoma (NSCLC) as it is considered a largely curable malignancy. Despite this, there are many unknowns regarding the future of healthcare in the US and legislative interventions addressing barriers to access to care for these patients. While the Affordable Care Act (ACA) presented many opportunities via an open marketplace for universal health insurance, it also presented a multitude of challenges [7]. Ongoing political dynamics make its future uncertain. Additionally, the repeal of the Sustainable Growth Rate (SGR) without an immediate replacement also presents its own issues in regard to Medicare reimbursement [8]. For example, while the US Congress extended a Medicare payment freeze to free-standing radiation oncology clinics up to December 2019, no long-term reimbursement plan exists [9]. Targeted health legislature and appropriate reimbursement rates are imperative, as they have been shown to have a direct impact on treatment patterns regardless of medical necessity [10]. Complicating these issues, there simply is not enough scientific literature to understand what defines equitable access to care and what interventions are effective [11].

This study hopes to identify the areas where primary prevention and access to care can be most impactful in early-stage NSCLC by reporting differential treatment and survival outcomes when stratified by urban versus rural geography.

## Materials and methods

Data Source

The Surveillance, Epidemiology, and End Results (SEER) 18 database of the National Cancer Institute (NCI) was used to obtain a case list of the stage-1 T1-2N0 NSCLC (side codes: 34.0-34.9) patients over the age of 60 who were diagnosed from 2004 to 2012. American Joint Commission on Cancer (AJCC) Staging Manual, 6th edition, was made available for these patients. Small-cell lung cancer or that of unknown histology was excluded (ICD codes: 8041-8045). We excluded patients for whom details regarding local treatment, staging, and/or age were not known. Patient demographics, treatment information, and survival outcomes were retrieved. Variables for which over 50% were not coded or unknown for any geographic group were not analyzed, in order to minimize bias (including sub-T stage and surgical approach). Chemotherapy-related information was not available for analysis. Survival was determined from the date of diagnosis to date of the last follow-up. All data were de-identified and made public for research purposes, and these were exempt from Institutional Review Board (IRB) approval process.

Urban-rural classification

The US Department of Agriculture's urban-rural continuum codes were used to classify patients into three general categories. Nine county- population categories exist based on location and population. They are as follows (in the order of most urban to most rural): (1) metropolitan areas where population is >1 million; (2) counties in metropolitan areas with a population of 250,000 to 1 million; (3) counties in metropolitan areas with a population of <250,000; (4) urban non-metropolitan areas with a population of >20,000 adjacent to a metropolitan area; (5) urban non-metropolitan counties with a population of >20,000 not adjacent to a metropolitan area; (6) urban non-metropolitan areas with a population of 2,500-19,999 adjacent to a metropolitan area; (7) urban non-metropolitan areas with a population of 2,500-19,999 not adjacent to a metropolitan area; (8) rural counties with a population of <2,500 adjacent to a metropolitan area; and (9) rural counties with a population of <2,500 not adjacent to a metropolitan area. For the purpose of this study, we categorized codes 1-3 as metropolitan (most urban), codes 4-7 as urban, and codes 8-9 as rural (most rural).

Statistical analysis

Pearson’s chi-squared test and independent t-test were used to determine the differences between categorical and continuous variables, respectively. Univariate and multivariate survival curves were derived using Kaplan-Meier actuarial methods stratified by rural, urban, and metropolitan populations. The model was built by sequentially adding all variables significant on univariate analysis and removing one-by-one to optimize overall statistical power. The Cox proportional hazards model was used to derive hazard ratios (HR) and their 95% confidence interval (CI) once fitting for covariates. Statistical Analysis System JMP (SAS-JMP) 14 (SAS Institute, Cary, NC) was utilized for statistical accuracy. Statistical significance was considered at an alpha of 5%.

## Results

Descriptive statistics

Urban and rural patients were more likely to be male, white, young, poor, and uninsured or Medicaid-dependent [probability value (p): <0.0001]. Early-stage NSCLC managed in rural and urban settings was more likely to be of squamous histology when compared to metropolitan settings (42.2%, 40.3%, and 31.2% respectively; p: <0.0001). Rural and urban patients were less likely to receive surgery compared to metropolitan patients (66%, 63%, and 67% respectively: p: <0.0001). The patient characteristics are reported below (Table 1).

**Table 1:** Patient characteristics (n = 62,213) SD: standard deviation; NOS: not otherwise specified

Category	Variables	Metropolitan (n = 53,647)	Urban (n = 7,483)	Rural (n = 1,031)	P-value
Gender	Female	27,521 (51.3%)	3,385 (45.2%)	422 (40.9%)	0.0001
	Male	26,126 (48.7%)	4,098 (54.7%)	609 (59.1%)	
Race	White	46,021 (85.8%)	6,845 (91.5%)	1,003 (97.3%)	0.0001
	Black	4,456 (8.3%)	466 (6.2%)	25 (2.42%)	
	Other	3,083 (5.8%)	164 (2.2%)	3 (0.3%)	
	Unknown	87 (0.2%)	8 (0.1%)	0 (0.0%)	
Age	60-69	18,285 (34.1%)	2,992 (40.0%)	432 (41.8%)	0.0001
	70-79	23,255 (43.4%)	3,252 (43.5%)	447 (43.4%)	
	80-89	11,491 (21.4%)	1,183 (15.8%)	150 (14.6%)	
	>90	616 (1.2%)	56 (0.8%)	3 (0.3%)	
Insurance status	Medicaid	3,077 (8.2%)	608 (11.6%)	94 (13.0%)	0.0001
	Insured	34,199 (91.1%)	4,588 (87.4%)	623 (85.8%)	
	Uninsured	265 (0.7%)	54 (1.0%)	9 (1.2%)	
Income	Mean +/- SD in 10s	4,884 +/- 1,050	3,304 +/-767	2,843 +/- 625	0.0001
Histology	Epithelial NOS	4,638 (8.7%)	774 (10.3%)	101 (9.8%)	0.0001
	Squamous	16,716 (31.2%)	3,012 (40.3%)	435 (42.2%)	
	Adenocarcinoma	16,716 (31.2%)	3,012 (40.3%)	435 (42.2%)	
	Cystic/mucinous/serous	1,112 (2.1%)	107 (1.4%)	11 (1.1%)	
	Acinar	731 (1.4%)	44 (0.6%)	3 (0.3%)	
	Complex epithelial	1,200 (2.2%)	169 (2.3%)	28 (2.7%)	
Treatment	Surgery	36,061 (67.2%)	4,739 (63.3%)	677 (65.6%)	0.0001
	Radiation	9,841 (18.3%)	1,522 (20.3%)	209 (20.3%)	
	Both	1,482 (2.7%)	233 (3.1%)	32 (3.1%)	
	Neither	6,263 (11.7%)	989 (13.2%)	113 (11.0%)	

Survival by location and treatment type

**Figure 1:**
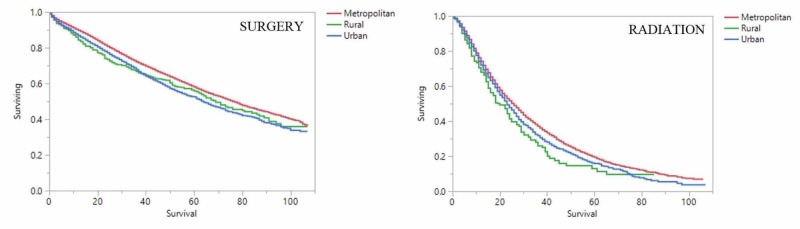
Kaplan-Meier overall survival by location and treatment X-axis: months surviving; Y-axis: proportion of patients surviving; a Kapan-Meier overall survival analysis for metropolitan (red), rural (green), and urban (blue) patients stratified by surgery (shown on right) and radiation (shown on left)

**Figure 2:**
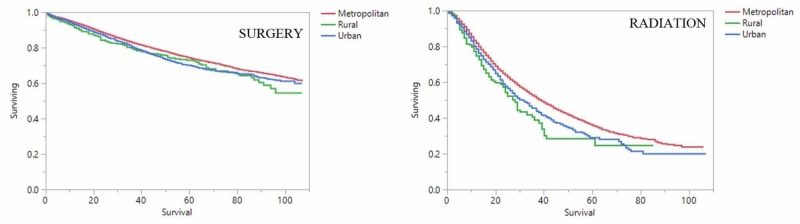
Kaplan-Meier cancer-specific survival by location and treatment X-axis: months surviving; Y-axis; proportion of patients surviving; a Kapan-Meier cancer-specific survival analysis for metropolitan (red), rural (green), and urban (blue) patients stratified by surgery (shown on right) and radiation (shown on left)

Metropolitan patients receiving surgery had the best overall survival (OS) at 2 years compared to urban and rural patients (81%, 77%, and 73% respectively; p: <0.0001). Similarly, metropolitan patients receiving radiation had superior OS compared to urban and rural patients (52%, 48%, and 41% at 2 years respectively; p: <0.0001). This holds up when accounting for causes of death as well. Metropolitan patients receiving surgery have superior cancer-specific survival (CSS) compared to urban and rural patients (88%, 86%, and 84% at 2 years respectively; p: <0.0001). Metropolitan patients receiving radiation have superior CSS compared to urban and rural patients (64%, 58%, and 54% at 2 years respectively; p: <0.0001). The above-mentioned findings are graphically represented here (Figures 1 and 2).

Multivariable analysis

Male gender, black race, radiation treatment, advanced age, squamous histology, and non-metropolitan geography were associated with worse OS (p: <0.0001) (Table 2). Upon censoring for non-lung cancer cause of death, male gender, black race, radiation treatment, advanced age, squamous histology, and non-metropolitan geography continued to be associated with worse survival rates (p: <0.0001) (Table 3). Cox proportional cancer-specific HRs for the geographical location of cancer care were 1.16 and 1.25 for urban and rural settings, respectively, when using metropolitan settings as a reference (p: <0.0001).

**Table 2:** Overall survival on multivariable analysis REF: reference variable; NOS: not otherwise specified

Category	Variables	Hazard ratio	P-value
Gender	Female	REF	<0.0001
	Male	1.36	
Race	White	REF	<0.0001
	Black	1.04	
	Other	0.82	
Treatment	Surgery	0.22	<0.0001
	Radiation	0.53	
	Both	0.38	
	Neither	REF	
Age	60-69	REF	<0.0001
	70-79	1.31	
	80-89	1.63	
	>90	2.02	
Histology	Epithelial NOS	0.95	<0.0001
	Squamous	REF	
	Adenocarcinoma	0.71	
	Cystic/mucinous/serous	0.78	
	Acinar	0.57	
	Complex epithelial	1.08	
Location of treatment	Metropolitan	REF	<0.0001
	Urban	1.15	
	Rural	1.21	

**Table 3:** Cancer-specific survival on multivariable analysis REF: reference variable; NOS: not otherwise specified

Category	Variables	Hazard ratio	P-value
Gender	Female	REF	<0.0001
	Male	1.31	
Race	White	REF	<0.0001
	Black	1.00	
	Other	0.86	
Treatment	Surgery	0.17	<0.0001
	Radiation	0.50	
	Both	0.38	
	Neither	REF	
Age	60-69	REF	<0.0001
	70-79	1.20	
	80-89	1.41	
	>90	1.74	
Histology	Epithelial NOS	1.00	<0.0001
	Squamous	REF	
	Adenocarcinoma	0.72	
	Cystic/mucinous/serous	0.85	
	Acinar	0.55	
	Complex epithelial	1.05	
Location of Treatment	Metropolitan	REF	<0.0001
	Urban	1.16	
	Rural	1.25	

## Discussion

While the relationship between low socioeconomic status and poor cancer-related outcomes is well-documented, the literature describing rural-urban disparities is scarce [12]. Previous studies have suggested that rural cancer patients are more likely to be diagnosed at an advanced stage, accounting for worse survival rates. However, in our stage-controlled cohort, the geographic area continued to play a significant factor in treatment utilization and survival [13]. Furthermore, these disparities persist in multivariate analysis despite controlling for age, race, gender, and treatment. A possible contributor unaccounted for in SEER is health status, which has been shown to be worse in rural areas when compared to urban areas [14]. However, this does not explain the inferior cancer-specific survival rates in rural areas.

Interestingly, one international review of rural-urban cancer outcomes found that the geographical location's influence on the nature and level of access to care does not necessarily translate into inherent disparity [15]. Urban-rural differences reflect inter-country differences in service availability, environmental conditions, and socioeconomic disadvantage, suggesting the need to focus on underlying social and structural causes of disparity. A dedicated study of rural communities in the US shows that specialty care and procedures require greater travel distance and time when compared to urban communities, presenting a concrete barrier to necessary care [16]. Our data corroborate these studies, as they show rural patients are less likely to receive optimal treatment for their otherwise curable lung-cancer diagnoses.

Oncologic resection is currently the standard of care for stage-1 NSCLC patients who are surgical candidates but requires hospitalization with multiple night-stay and medical candidacy [17]. Stereotactic body radiotherapy (SBRT) is a non-invasive procedure generally reserved for patients too sick to undergo surgery. However, patients likely to forgo treatment should also receive a referral for SBRT as clinical cures have been achieved by as little as one ablative-dose radiation treatment. This may be considered for patients who refuse surgery otherwise as well [18]. Because the distance to surgery and radiation centers is a factor in obtaining definitive treatment for rural cancer patients, improved prevention and screening must be met by interventions that aim to improve access to definitive surgery or radiotherapy [19].

The SEER database is subject to many limitations. While we were unable to account for biases inherent to a retrospective study, the large sampling size and population-based sampling methods allowed us to best estimate actual differences. Some patients received both surgery and radiation, the rationale for which is not clear. We hypothesize this is due to suboptimal resection (i.e., positive margins) and/or pathological upstaging not discernible in SEER. Details about patient comorbidity, chemotherapy, and radiation modality/dose were not available. As such, many patients categorized as receiving radiation treatment likely received palliative conventional dosing as opposed to the SBRT given its relatively recent adoption into the National Comprehensive Cancer Network (NCCN) guidelines [17]. In the future, as the use of SBRT technique increases, population-based local control and survival with definitive radiotherapy for early-stage NSCLC are expected to improve significantly.

## Conclusions

Non-metropolitan early-stage NSCLC patients are more likely to harbor high-risk features of the disease and are less likely to receive definitive treatment. Adoption of lung-cancer screening guidelines must be accompanied by interventions in primary prevention and improvements to geographic access to care, as this will enable the diagnosis to shift to an earlier (and curable) stage where multiple treatment options are available. 
